# The Rice Oligonucleotide Array Database: an atlas of rice gene expression

**DOI:** 10.1186/1939-8433-5-17

**Published:** 2012-07-19

**Authors:** Peijian Cao, Ki-Hong Jung, Daeseok Choi, Daehee Hwang, Jun Zhu, Pamela C Ronald

**Affiliations:** Institute of Bioinformatics, Zhejiang University, Hangzhou, 310058 China; Department of Plant Molecular Systems Biotechnology & Crop Biotech Institute, Kyung Hee University, Yongin, 446-701 Korea; School of Interdisciplinary Bioscience and Bioengineering & Integrative Biosciences and Biotechnology, POSTECH, Pohang, 790-784 Korea; Department of Plant Pathology and the Genome Center, University of California, Davis, 95616 USA; Joint Bioenergy Institute, Emeryville, 94710 USA

**Keywords:** Rice oligonucleotide array database, Gene expression analysis, Meta-analysis, Co-expression, GO enrichment analysis

## Abstract

**Background:**

Microarray technologies facilitate high-throughput gene expression analysis. However, the diversity of platforms for rice gene expression analysis hinders efficient analysis. Tools to broadly integrate microarray data from different platforms are needed.

**Results:**

In this study, we developed the Rice Oligonucleotide Array Database (ROAD,http://www.ricearray.org) to explore gene expression across 1,867 publicly available rice microarray hybridizations. The ROAD’s user-friendly web interface and variety of visualization tools facilitate the extraction of gene expression profiles using gene and microarray element identifications. The ROAD supports meta-analysis of genes expressed in different tissues and at developmental stages. Co-expression analysis tool provides information on co-regulation between genes under general, abiotic and biotic stress conditions. Additionally, functional analysis tools, such as Gene Ontology and KEGG (Kyoto Encyclopedia of Genes and Genomes) Orthology, are embedded in the ROAD. These tools facilitate the identification of meaningful biological patterns in a list of query genes.

**Conclusions:**

The Rice Oligonucleotide Array Database provides comprehensive gene expression profiles for all rice genes, and will be a useful resource for researchers of rice and other grass species.

**Electronic supplementary material:**

The online version of this article (doi:10.1186/1939-8433-5-17) contains supplementary material, which is available to authorized users.

## Background

Rice (*Oryza sativa*) is a staple food for more than 50% of the human population. Because of the high level of genomic colinearity and conservation of gene function among grass species, rice serves as a useful research model in other grass studies (Devos and Gale[2000]; Jung et al.[2008a]). The complete sequencing of rice genome achieved in year 2005 (International Rice Genome Sequencing Project[2005]), has brought biological research to the genome scale and post-genome era, while assigning function to every rice gene is still an enormous challenge. Comprehensive annotations of rice genome sequence have revealed that more than half of the predicted genes do not have assigned biological functions (Yuan et al.[2005]; Itoh et al.[2007]; Tanaka et al.[2008]). Despite extensive efforts to characterize the function of rice genes, only a handful of biological functions have been identified, mostly through the laborious process of map-based cloning (Jung et al.[2008a]).

Microarray technologies are an important strategy for genome-wide expression pattern analysis and is becoming increasingly important for gene functional analysis (Schmid et al.[2005]). Several rice array platforms for the two rice subspecies (ssp. *japonica* and *indica*) have been reported and their characteristics are summarized in Table 1. The GeneChip rice genome array, designed by Affymetrix and produced using a direct synthesis method, contains 57,381 probesets covering approximately 48,564 and 1,260 transcripts from the *japonica* and *indica* cultivars, respectively. Agilent has constructed a 22K Rice Oligo Microarray Kit based on rice FLcDNAs and recently announced a 44K version (Shimono et al.[2007]). The *Oryza sativa* Genome Oligo Set (Version 1.0; 61K) was designed by the Beijing Genomics Institute and Yale University (BGI/Yale) and based on the draft *indica* and *japonica* sequences. The University of California, Davis, led a National Science Foundation (NSF) supported effort to design, print and validate 22K and 45K oligonucleotide arrays based on gene model predictions from TIGR’s osa1 version 3.0 release.

**Table 1:** **Summary of rice microarray platforms available in ROAD**

Platform	No. of probes (oligo length, nt)	MSU RGAP V6		RAP V3		KOME cDNA		No. of experiments/samples in ROAD
		No. of matched probes	No. of matched loci/gene models	No. of matched probes	No. of matched loci/gene models	No. of matched probes	No. of matched cDNAs	
Affymetrix	57,381 (25)	44,399	37,993/47,652	33,070	27,778/34,559	30,690	34,438	62/1,155
Agilent 22K	22,575 (60)	16,864	15,974/22,774	18,853	18,010/20,883	21,393	24,284	19/104
Agilent 44K	45,220 (60)	35,287	24,259/33,301	35,928	24,467/31,643	34,512	32,092	12/77
BGI/Yale	60,727 (70)	35,269	31,949/40,904	29,042	25,182/30,581	26,645	30,076	6/322
NSF 20K	21,120 (50-70)	18,156	18,150/23,489	12,697	13,040/15,879	10,982	15,311	2/120
NSF 45K	43,311 (50-70)	39,372	37,792/46,251	22,996	22,608/27,223	19,338	24,956	4/89

These rice microarray platforms have been successfully used in characterizing gene expression profiles from different tissues and organs (Wang et al.[2010]), different cell types (Jiao et al.[2009]), under biotic and abiotic treatment conditions (Jung et al.[2008b]; Swarbrick et al.[2008]; Jung et al.[2010]), identification of alternative splice (Jung et al.[2009]) and mutants (Bruce et al.[2009]). As a result, an increasing number of rice microarray datasets are being deposited in public repositories such as the Gene Expression Omnibus (GEO) at the National Center for Biotechnology Information (NCBI) (Barrett et al.[2009]), the ArrayExpress at the European Bioinformatics Institute (EBI) (Parkinson et al.[2007]) and the Center for Information Biology gene EXpression database (CIBEX) at the DNA Data Bank of Japan (DDBJ) (Ikeo et al.[2003]). There are also several databases that allow for efficient access and data mining of collections of microarray data for rice (Table 2). For example, the Rice Expression Profile Database (RiceXPro,http://ricexpro.dna.affrc.go.jp/), which is based on the Agilent 44K microarray, provides an overview of the spatiotemporal gene expression profiles of various organs and tissues (Sato et al.[2011]). Genevestigator (https://www.genevestigator.ethz.ch/) provides a meta-analysis toolbox to explore gene expressions across a wide variety of biological contexts for rice and other species, but it is commercial and not completely publicly available (Hruz et al.[2008]). Other databases including OryzaExpress (Hamada et al.[2011]), RicePLEX within the Plant Expression Database (PLEXdb) (Dash et al.[2012]), Bio-Array Resource for Plant Biology (BAR) (Toufighi et al.[2005]) and Yale Virtual Center for Cellular Expression Profiling of Rice (Jiao et al.[2009]) are useful for expression pattern analysis of rice genes. Although general agreement between different microarray platforms has been shown to be low, data derived from high signal intensities can correlate between different platforms as well as in replicates of the same platform, and that overlap between significant gene lists from different platforms was as high as 67% when low intensity values were removed from an *Arabidopsis* study (Pylatuik and Fobert[2005]). This suggests the potential application for the broad integration of microarray data from different platforms. Here we describe the Rice Oligonucleotide Array Database (ROAD,http://www.ricearray.org), which integrates the most comprehensive public microarray datasets and provides several functional analysis tools. With a user-friendly web interface, the ROAD is a useful reference for elucidating rice gene expression and function.

**Table 2:** **Summary of databases for expression analysis of rice genes**

Database	Platform	Expression type	Co-expression	Output type	Other features	Reference
ROAD	- Affymetrix			- Heatmap	- Single/multiple-platform probe search	This study
	- Agilent 22K/44K	- Anatomy	- Whole dataset	- Line plot	- GO/KO enrichment analysis	
	- NSF 20K/45K	- Development	- Biotic stress	- Bar plot	- Meta-profiling analyses for both Affymetrix and Agilent 44K	
	- BGI/Yale	- Individual dataset	- Abiotic stress	- Network	- Accept gene list and bulk download	
				- Table		
PLEXdb	- Affymetrix	- Individual dataset	- N/A	- Heatmap	- Identify probes of homologs between different species	(Dash et al.[2012])
				- Line plot	- Rice and 15other plant species	
				- Bar plot		
				- Table		
Genevestigator	- Affymetrix	- Anatomy	- N/A	- Scatter plot	- Bi/hierarchical clustering	(Hruz et al.[2008])
		- Development		- Heatmap	- Accept gene list	
		- Perturbations			- Result export is prohibited	
		- Neoplasms			- Rice and 14 other organisms	
		- Individual dataset				
RiceXPro	- Agilent 44K	- Anatomy	- Anatomy	- Heatmap	- Provide useful web links for other analyses such as Rice TOGO, SALAD, RAP-DB	(Sato et al.[2011])
		- Development of leaf	- Development	- Bar plot	- Hierarchical clustering	
		- Root cell-type anatomy	of leaf	- Line plot	- *T*-test and fold change (FC) analysis	
					- Accept gene list	
BAR	- Affymetrix	- Individual dataset	- N/A	- Electronic fluorescent pictograph	- Rice and 6 other plant species	(Toufighi et al.[2005])
				- Table		
gAtlas in RiceGE	- Affymetrix	- Whole dataset	- N/A	- Bar plot	- Filter option to select specific Dataset	(Jung et al.[2011])
	- RMOS					
RiceNet in PlaNet	- Affymetrix	- Whole dataset	- Whole dataset	- Line plot	- Mapman ontology analysis	(Mutwil et al.[2011])
				- Network	- Provide phenotypes in *Tos17* insertion mutant lines	
				- Table	- Provide cluster network and node vicinity network separately	
OryzaExpress	- Affymetrix	- Whole dataset	- Whole dataset	- Line plot	- Diverse options for developing co- expression network (CA, PCC, MR and PAC)	(Hamada et al.[2011])
	- Agilent 22K			- Bar plot	- Predicted orthologs and networks in Arabidopsis	
				- Network	- 11 options to display data in network	
				- Table		
Gene Co-expression Network Browser	- Affymetrix	- N/A	- Whole dataset	- Network	- 45 gene modules	(Ficklin et al.[2010])
					- 76 enriched co-functional clusters	
					- GO/KO/IPR enrichment analysis	
					- 6 options to display data in network	
					- Rice and maize	

Note: Hyperlinks for the mentioned databases are as followings: ROAD (Rice Oligonucleotide Array Database),http://www.ricearray.org/; PLEXdb (Plant Expression Database),http://www.plexdb.org/; Genevestigator,https://www.genevestigator.com/gv/; RiceXPro,http://ricexpro.dna.affrc.go.jp/; BAR (The Bio-Array Resource for Plant Biology),http://bar.utoronto.ca/; gAtlas in RiceGE (Rice Functional Genomic Express Database),http://signal.salk.edu/cgi-bin/RiceGE?JOB=APPENDIX&QUERY=GeneAtlas; RiceNet in PlaNet,http://aranet.mpimp-golm.mpg.de/ricenet; OryzaExpress,http://bioinf.mind.meiji.ac.jp/OryzaExpress/; Gene Co-expression Network Browser,http://www.clemson.edu/genenetwork/.

## Results and discussion

### Microarray element search for multiple array platforms in rice

We collected information from six rice microarray platforms, including the Affymetrix, Agilent 22K and 44K, BGI/Yale, and the NSF 20K and 45K, to construct the ROAD. Probe sequences from each platform were extracted and mapped onto cDNAs to match the probes to the expressed genes; the cDNAs were drawn from the Rice Genome Annotation Project (RGAP, previously TIGR) V6 (Ouyang et al.[2007]), Rice Annotation Project (RAP) V3 (Tanaka et al.[2008]) and the Knowledge-based Oryza Molecular biological Encyclopedia (KOME) (Kikuchi et al.[2003]) (Table 1). Because microarray platforms use their own element IDs rather than common gene IDs, two search tools were developed to determine the relationship between microarray elements and rice genes. The ‘Single-platform Microarray Element Search’ and the ‘Multiple-platform Microarray Element’ search tools allow users to identify the specific platform probes that map to a common gene target. Users may choose between entering a list of IDs (genes or microarray elements) of interest and uploading a file to use these search tools. The search result can be returned as HTML format for browsing or txt format for download. The entire probe mapping matrix table is also available for download. ‘Single-platform Microarray Element Search’ tool in ROAD is similar with ID converter tool in OryzaExpress which provides the RAGP IDs, RAP IDs and Affymetrixprobe IDs for querying and cross-linking (Hamada et al.[2011]), while probe IDs from six array platforms corresponding to RAGP, RAP and KOME IDs are provided in ROAD.

### Gene expression analysis

**Figure 1:**
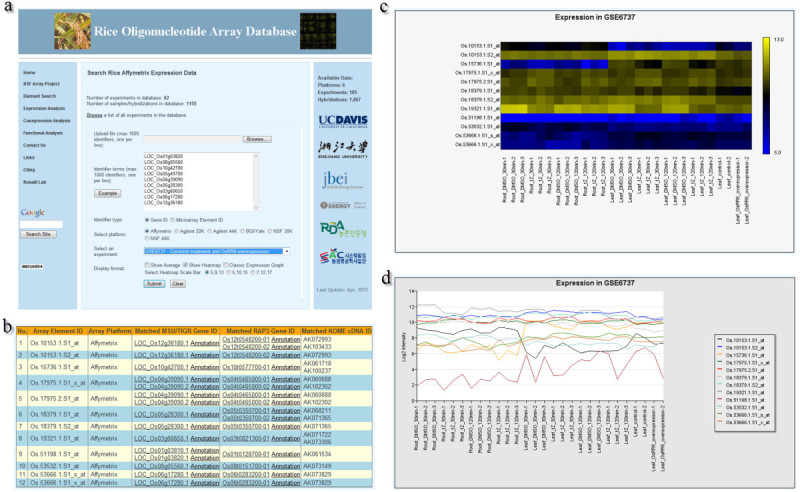
**Process of gene expression profiling analysis in ROAD.** (**a**) A snap shot of the web interface for gene expression search in ROAD. Gene expression search can be initiated by entering one (or multiple) gene(s) or microarray element ID(s), such as RGAP ID, RAP ID, KOME ID, Affymetrixprobeset ID, or by uploading a file containing them. Then select the microarray platform and specific experiment to search against and the display format. (**b**) Tabular list of microarray elements mapped onto the example gene IDs when selecting Affymetrix platform. (**c**) Heatmap representation of expression profiles retrieved by the example genes from Affymetrix experiment GSE6737. (**d**) The classical line plot representation of the same data with (**c**).

Raw rice microarray data from six platforms (including 105 experiments and 1,867 hybridizations from March 2012, electronic Additional file1: Table S1) were downloaded from public repositories and were normalized using the same method. One-channel and two-channel platforms were normalized separately because of differences in platform features or designs (MAS 5.0 for one-channel platform, Lowess and MAD for two-channel platforms). For two-channel platforms, the normalized expression ratios (log_2_(Cy3/Cy5)) were used and the color-swap hybridizations were manually corrected to make them comparable among other samples. Normalized data were integrated into the ROAD, thus simplifying the retrieval process of their gene expression profiles. After entering a list of genes or microarray element IDs into the ROAD, the user can then select the microarray platform and specific experiment to search against (Figure1a). The “Gene Expression Search” tool in ROAD provides a tabular list of microarray element IDs and of matching gene IDs from RGAP, RAP and KOME (Figure1b). Clicking on the gene IDs will redirect users to RGAP and RAP-DB databases to obtain detailed information on gene annotation. The expression profiles for query genes can be shown either as a heatmap (Figure1c) or a classic line plot (Figure1d). For one-channel platform Affymetrix, heatmap representation is generated by Blue-Black-Yellow color scheme, while Green-Black-Red for two-channel platforms (Agilent 22K and 44K, BGI/Yale, NSF 20K and 45K). The scale bar for the heatmap output can be adjusted using several options. Because there are multiple microarray elements matched for some genes, the heatmap and line plot are displayed according to microarray element IDs. Besides displaying expression profile from multiple biological or technical replicates within an experiment, the average expression of replicates can also be generated when checking “Show Average” checkbox. The download option allows users to easily transfer expression data into other databases or software for further analysis. The experiments integrated into ROAD can be searched by the “Experiment Search” tool and the “Highly Expressed Genes” tool allows users to quickly identify a set of genes that are highly expressed under a selected expression threshold in a specific tissue.

### Meta-analysis on anatomy and development from Affymetrix and Agilent 44K platforms

**Figure 2:**
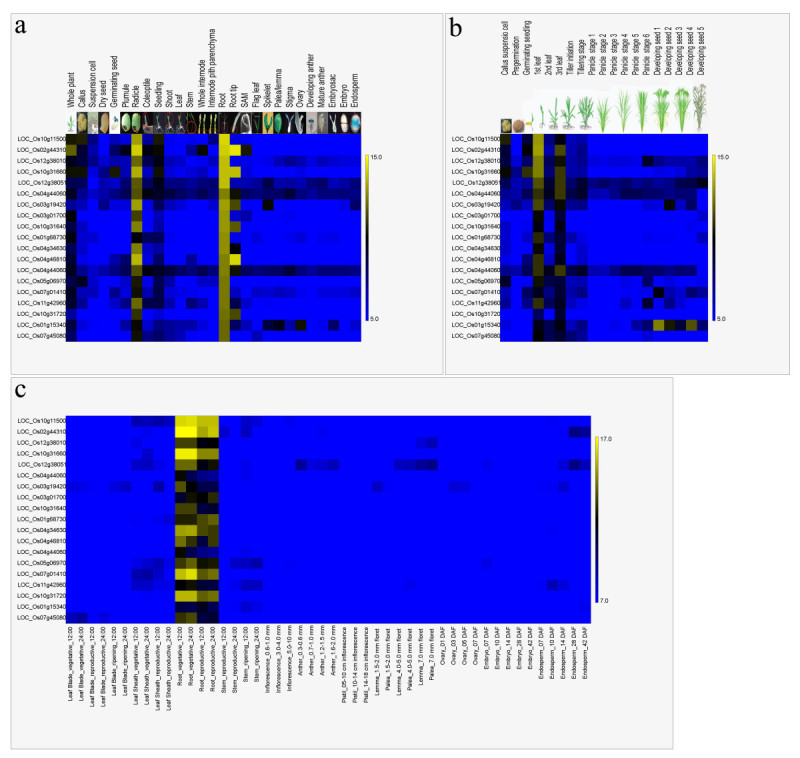
**Screenshots of a ROAD meta-analysis using Affymetrix (a and b) and Agilent 44K (c) array platforms.** Nineteen genes that are preferentially expressed in root were selected and used for the three meta-analyses. (**a**) Meta-analysis query of diverse tissues. (**b**) Meta-analysis query of diverse developmental stages. (**c**) Meta-analysis query of diverse floral and vegetative organs.

Because many factors such as RNA isolation, labeling and hybridization methods affect the quality of microarray data, pooling data from different experiments does not allow for a rigorous expression profiling analysis. Genevestigator developed a novel approach (meta-analysis) to assemble microarray data from different experiments into context-related profiles (meta-profiles). This large-scale combination and analysis of expression data from a single organism using a single platform allows the identification of biologically meaningful expression patterns of individual genes (Hruz et al.[2008]). One drawback to Genevestigator, however, is that the public has limited access to the platform’s functions. As the open access version only supports the meta-analysis of a maximum of 50 genes at one time and does not allow data downloading, further analysis of the gene expression data is hindered. Therefore, we constructed a meta-analysis tool based on the 1,155 Affymetrix hybridizations and 209 Agilent 44K hybridizations found in the ROAD. This construction was possible because both the Affymetrix and Agilent 44K platforms provide standardized systems with a high degree of reproducibility. We have also developed four meta-profiles for genes expressed in different tissues and at different developmental stages for each of the two platforms. In case of the meta-profiles for the developmental stages, the Affymetrix meta-profile provides the average gene expression levels in all tissues during one developmental stage. The Agilent 44K provides the average gene expression levels in leaf blades during 17 developmental stages. Through analyzing Affymetrix and Agilent 44K anatomic meta-profiles, 19 root-preferential genes were identified and their anatomic (Figure2a and2c; Electronic Additional file2: Figure S1a and c) and developmental expression patterns (Figure2b; Electronic Additional file2: Figure S1b, d and e) on both platforms are shown in Figure2 and electronic Additional file2: Figure S1. This meta-analysis allowed us to evaluate root-preferential expression patterns. The meta-profile developmental stage analysis from the Affymetrix array platform indicates that these 19 genes were preferentially expressed during seedling stages (Figure2b and electronic Additional file2: Figure S1b). Using the Agilent 44K platform, we found that expression levels in leaf blades were lower than those in the root (Electronic Additional file2: Figure S1d and e). These results show that by integrating data for different anatomic tissues and developmental stages, the meta-analysis tool provides straightforward information about where and when genes of interest are expressed.

### Co-expression analysis

Similarity of gene expression profiles (co-expression) can provide powerful information to identify new genes functionally related. The rapid accumulation of microarray data in past decade allows the creation of co-expression networks by examining the co-expression patterns of genes over a large number of experimental conditions. Several online co-expression analysis tools have been developed for rice, including OryzaExpress (Hamada et al.[2011]), RiceArrayNet (Lee et al.[2009]) and ATTED-II (Obayashi et al.[2011]). During co-expression analysis, more microarray data will generate better reliability. Based on the collection of the most comprehensive microarray data in ROAD (Table 2), we also developed co-expression analysis tools in ROAD. After filtering out of 74 outliers and selecting hybridizations involved in abiotic and biotic stresses, three kinds of co-expression relationships between rice genes were calculated, including general co-expression, abiotic and biotic stress co-expression, based on 1,081, 329 and 181 Affymetrix microarray hybridizations individually. The widely used Pearson correlation coefficient (PCC) index was selected to evaluate the similarities of expression profiles for gene pairs. We selected default values of the PCC cutoff as 0.75 and 0.8, for general and abiotic and biotic stresses, respectively. At this relatively stringent cutoff value, the general or abiotic and biotic stress co-expression networks can be constructed by using online network drawing tool. After entering a list of guide-genes and selecting the network type, a co-expression network will be generated by Cytoscape Web, an interactive web-based network browser (Lopes et al.[2010]). The network can be easily zoomed in/out and external RGAP database link is provided for each node. The download option allows users to export the network into local file with SIF format which can be used in local Cytoscape or other network analysis software. Lower co-expression under the selected PCC cutoff values and negative co-expression may still be meaningful for some genes, so we developed another tool to extract positively and negatively co-expressed genes with query gene under a user entered PCC cutoff, not limited to the cutoff values used in network construction. All the current rice co-expression analysis tools in Table 2 use the whole microarray dataset to construct the network except ROAD and RiceXPro. Of them, RiceXPro provides two co-expression profiles such as anatomy (spatiotemporal gene expression of various tissues/organs across entire life cycle) and development of leaves (Sato et al.[2011]). The abiotic and biotic co-expression network tools in ROAD will provide useful information for elucidating the relationship of stress related genes. It has been proven to be reasonable to calculate the co-expression for each set of diverse experiment conditions with a clear biological meaning in ATTED-II (Obayashi et al.[2011]), supporting the possible functionality of rice abiotic and biotic co-expression network tools.

### Functional analysis using gene ontology or KEGG orthology

Gene Ontologies (GO) provide controlled vocabulary to describe the biological process, molecular function, and component of the cell to which a gene product putatively contributes (Ashburner et al.[2000]; Berardini et al.[2004]; The Gene Ontology Consortium[2008]). KEGG Orthology (KO) consisting of manually defined ortholog groups that correspond to KEGG pathway nodes and BRITE hierarchy nodes, is the basis for the representation of KEGG reference pathway maps and BRITE functional hierarchies (Kanehisa et al.[2010]). GO and KO analyses are useful for identifying biological patterns in a list of genes, microarray datasets, or cDNA collection. For example, GO enrichment analysis has been successfully applied to assessment of rice light-responsive genes (Jung et al.[2008b]). To facilitate GO and KO analyses of query genes, we developed online tools to identify the enriched or depleted GO/KO terms within a query gene list based on a hypergeometric distribution. These tools provide a tabular list of GO/KO terms mapped onto the query genes with detailed information for each term. Next, a hypergeometric *p* value is calculated for each GO/KO term, whose value is based on comparisons of the observed number from the queried gene list and the expected number from the genome scale.

## Conclusions

The Rice Oligonucleotide Array Database is designed to provide a comprehensive gene expression profile for all rice genes. Our current meta-analysis tool focuses on expressions specific to tissue and developmental stages. This analysis will be expanded to include meta-profiles of genes expressed during the rice response to abiotic and biotic stresses and to hormone treatment. New microarray data will be normalized and imported into ROAD semi-automatically on a regular. We anticipate that this database will be useful to researchers of rice and other grass species and that it will accelerate the identification of gene function in monocotyledonous species.

## Methods

### Microarray data and database construction

As of March 2012, microarray data from 105 rice microarray experiments (1,867 hybridizations) were collected from NCBI GEO (Barrett et al.[2009]), EBI ArrayExpress (Parkinson et al.[2007]) and PLEXdb (Dash et al.[2012]). The raw data was downloaded and experiments without raw data were discarded. For one-channel array (Affymetrix), MAS 5.0 method provided by the R package, affy, for the Affymetrix rice array was used to conduct background correction, normalization, probe specific background correction, probe summarization and convert probe level data to expression values (Affymetrix[2012]). The trimmed mean target intensity of each array was arbitrarily set to 500. The data were then log_2_ transformed. For two-channel arrays (Agilent 22K and 44K, BGI/Yale, NSF 20K and 45K), R package marray in Bioconductor was used to do the normalization with within-array Lowess and between-array MAD scale normalization methods (Cleveland[1979]; Wang et al.[2002]). The color-swap hybridizations were manually corrected to make them comparable among other samples. In case of Agilent 44K array data used for meta-profiling analyses, we converted the median signal intensities of Cy3 to log_2_ median intensities and then normalized the log_2_ intensities using the quantile normalization method (Bolstad et al.[2003]). The sequences of probes were extracted from each platform website and then mapped onto RGAP V6, RAP V3 and KOME cDNAs using NCBI Blast with 100% identity over 100% coverage (Altschul et al.[1990]). Regarding Affymetrixprobeset which have 11 probe pairs, the probeset with at least half perfect-match (PM) probes matched onto cDNA sequence was considered as mapped.

The ROAD database was constructed with PHP (Hypertext Preprocessor) and MySQL, run on a Windows 2003 server. The http address ishttp://www.ricearray.org. Heatmap and classic line plots were generated by the PHP library JpGraph (http://jpgraph.net/).

### Co-expression analysis

After MAS normalization of all Affymetrix microarray samples, outliers were detected using the arrayQualityMetricsBioconductor package, which uses three different statistical tests to identify outliers (Gentleman et al.[2004]; Kauffmann et al.[2009]). Seventy-four samples failed at least one test and were considered as outliers and removed from the dataset. As a result, a total of 1,081 Affymetrix samples remained for co-expression analysis. For genes with multiple Affymetrixprobesets matched, the probeset with highest expression profile was used. There are several kinds of methods to evaluate the strength of co-expression, such as Pearson correlation coefficient (PCC), mutual rank (MR) based on rank transformations of the weighted PCC (Obayashi and Kinoshita[2009]) and correspondence analysis (CA) (Yano et al.[2006]). Although PCC takes a long-calculation time and was considered to contain many false-positives (Hamada et al.[2011]; Obayashi et al.[2011]), it has been widely used as an index in the co-expression analysis, such as RiceArrayNet (Lee et al.[2009]), RiceXPro (Sato et al.[2011]) and Gene Co-expression Network Browser (Ficklin et al.[2010]). The success in functional study of plant genes using PCC has also been reported (Fujii et al.[2010]; Matsuura et al.[2010]; Soeno et al.[2010]). Therefore, we adopted PCC to measure tendency of co-expression between genes based on these 1,081 Affymetrix samples. To choose an appropriate PCC cutoff value to construct co-expression network, we examined the changes in the node number, edge number, and network density as a function of PCC cutoff values. As the cutoff value increased, both the node number and edge number decreased; however, as the cutoff reached a relatively high value, the decreasing rate of edges became slower than that of nodes, which might lead to an increase in the network density. Indeed, the network density showed minima around 0.75 (general) and 0.8 (abiotic and biotic stresses) PCC cutoff values and increased thereafter. Therefore, we selected default values of the PCC cutoff as 0.75 and 0.8, for general and abiotic and biotic stresses, respectively. Cytoscape Web, an interactive web-based network browser, was used as the network viewer (Lopes et al.[2010]).

### GO and KO enrichment analysis

The GO terms and assignments for rice genes were downloaded from Gramene database (http://www.gramene.org/) (Jaiswal[2011]) and KO from KEGG database (http://www.genome.jp/kegg/) (Kanehisa et al.[2010]). The RAP rice gene locus IDs in KEGG database were converted to RGAP IDs using RAP-DB ID converter tool (http://rapdb.dna.affrc.go.jp/tools/converter) (Tanaka et al.[2008]). Then hypergeometric distribution was used to calculate the *p* value for GO and KO enrichment analyses.

## Electronic supplementary material

Additional file 1: **Table S1.** Detailed information of rice microarray experiments available in ROAD. (XLS 204 KB)

Additional file 2: **Figure S1.** Screenshots of meta-analysis in ROAD queried with 19 root-preferential genes for anatomy (a) and developmental stages (b) of Affymetrix array platform, and anatomy (c) and developmental stages (d, e) of Agilent 44K array platform. (JPEG 5 MB)

Below are the links to the authors’ original submitted files for images.

Authors’ original file for figure 1

Authors’ original file for figure 2
