# Supplementary figures and images for: The Rice Oligonucleotide Array Database: an atlas of rice gene expression
Source: Rice (N Y). 2012 Jul 19;5:17. doi: 10.1186/1939-8433-5-17 (PMC4883718; doi:10.1186/1939-8433-5-17)

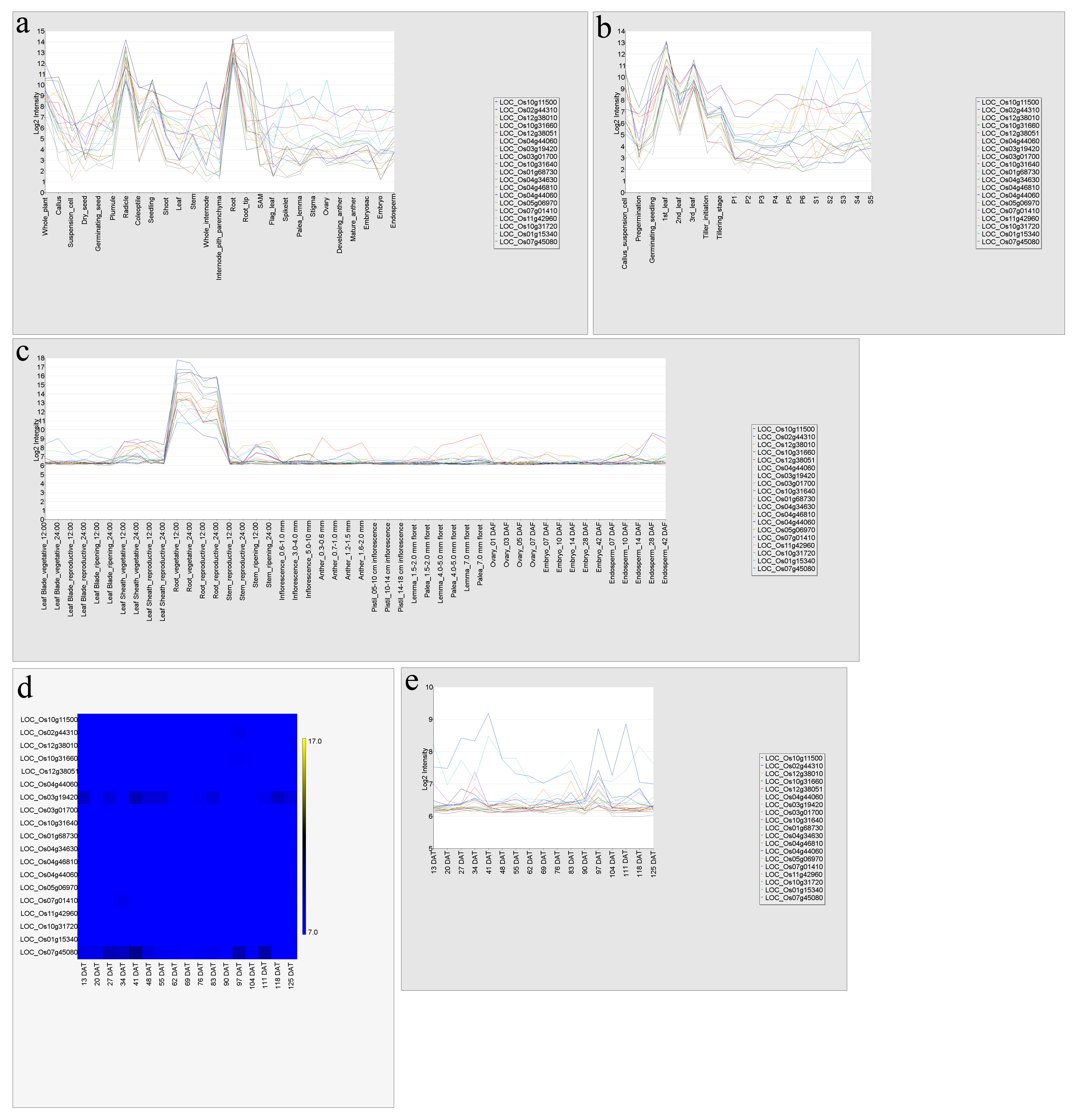

Supplement: Supplementary file 2 — Additional file 2: Figure S1. Screenshots of meta-analysis in ROAD queried with 19 root-preferential genes for anatomy (a) and developmental stages (b) of Affymetrix array platform, and anatomy (c) and developmental stages (d, e) of Agilent 44K array platform. (JPEG 5 MB) [file 12284_2012_27_MOESM2_ESM.jpeg]

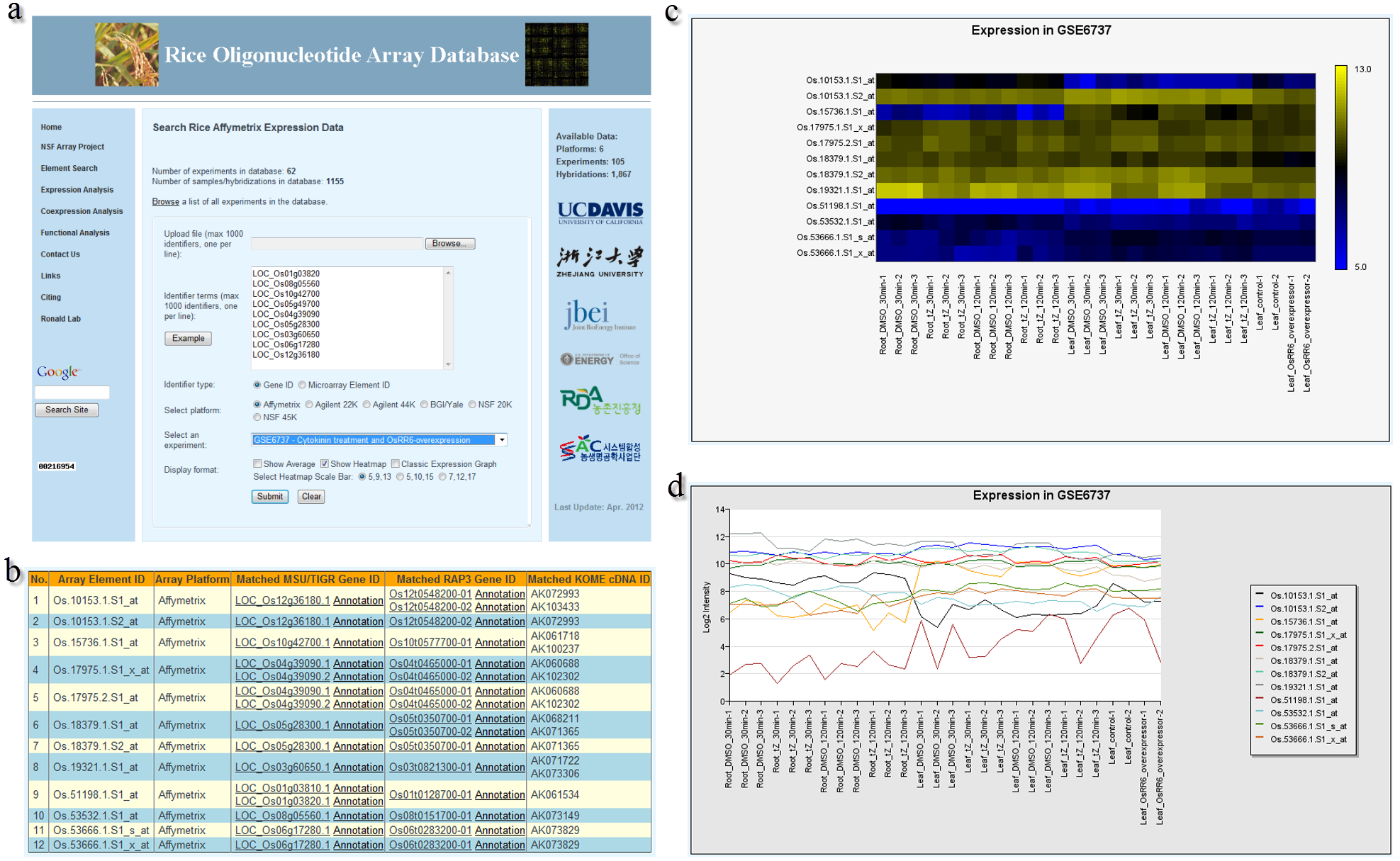

Supplement: Supplementary file 3 — Authors’ original file for figure 1 [file 12284_2012_27_MOESM3_ESM.png]

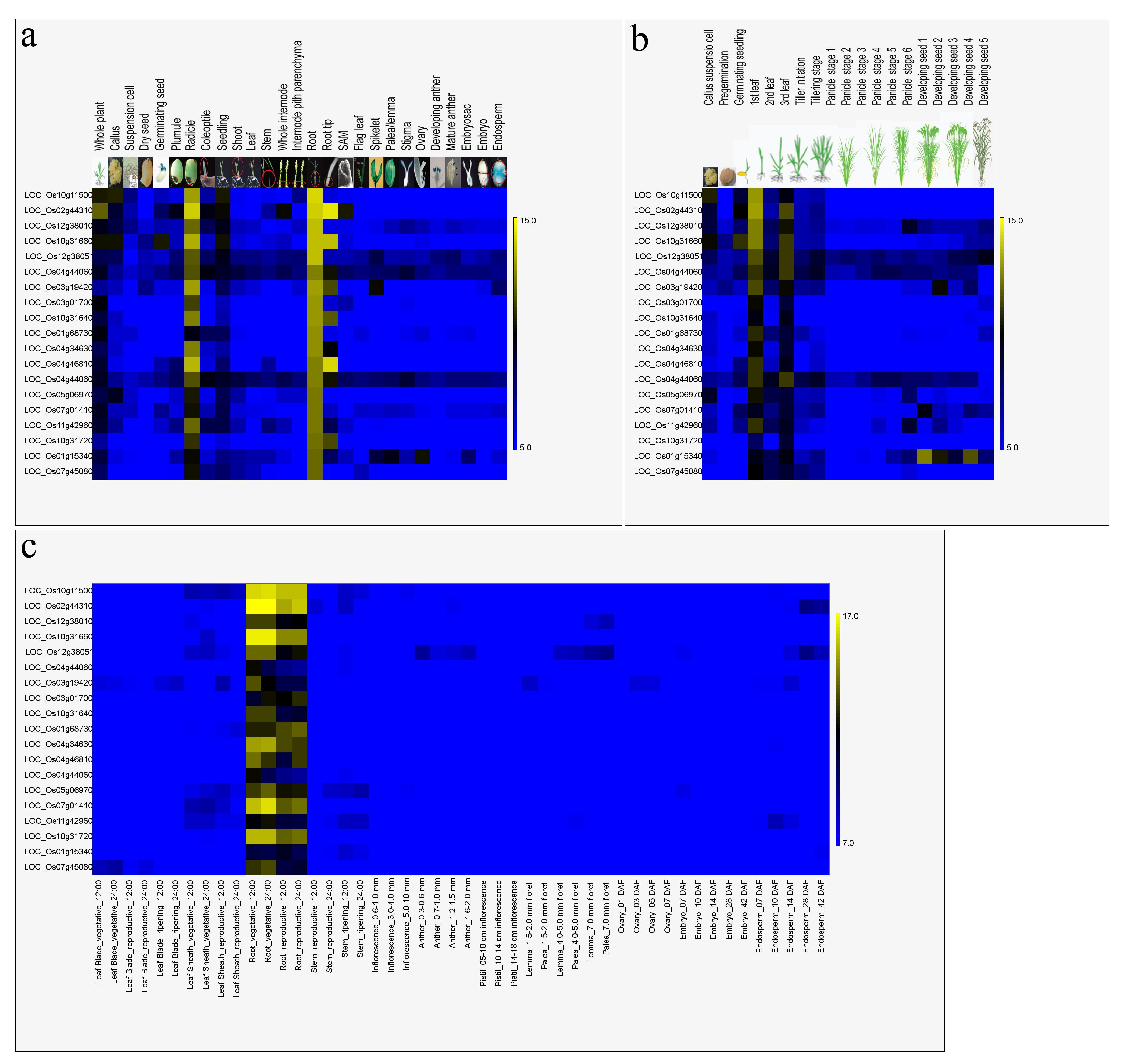

Supplement: Supplementary file 4 — Authors’ original file for figure 2 [file 12284_2012_27_MOESM4_ESM.jpeg]
